# Perspectives of Child Life Specialists After Many Years of Working With a Humanoid Robot in a Pediatric Hospital: Narrative Design

**DOI:** 10.2196/23496

**Published:** 2020-11-19

**Authors:** Tanya Beran, Jacqueline Reynolds Pearson, Bonnie Lashewicz, Sandy Baggott

**Affiliations:** 1 Department of Community Health Sciences University of Calgary Calgary, AB Canada; 2 Alberta Children's Hospital Calgary, AB Canada; 3 University of Calgary Calgary, AB Canada

**Keywords:** child life, support, pediatric, distraction, robotics, human-robot interaction

## Abstract

**Background:**

Child life specialists (CLSs) play an important role in supporting patients and their families during their visits to a children’s hospital. Although CLSs are equipped with considerable expertise to support families during some of the most difficult moments of their lives, we introduced an additional resource to them in the form of a humanoid robot named MEDi.

**Objective:**

The aim of this study is to explore the experiences of CLSs using a robot to support children.

**Methods:**

We interviewed 7 CLSs who had worked with this robot for several years. The transcribed interviews were analyzed using open and axial coding.

**Results:**

The first main theme that emerged was the process of navigating from fear to friendship in learning to use a humanoid robot for therapeutic support. The second major theme was MEDi as a source of connection and support to children. CLSs’ perceptions of MEDi as an adaptable resource and working with the limits of MEDi constituted the last 2 themes.

**Conclusions:**

These descriptions show how CLSs can incorporate a robot into their practice.

## Introduction

### Overview

Child life specialists (CLSs), also known as play specialists and child life therapists in various countries, work toward creating a friendly hospital environment to facilitate children’s healing [1]. The role of CLSs is multifaceted, whereby professionals with university education, including internship experience, help infants, children, youth, and families cope with illness, injury, and treatment [2]. The broad scope of the CLS role entails supporting people with a range of challenges, needs, and backgrounds every day. Despite the many tools in the form of play, behavior management strategies, and education [3], providing support to sick children and their families, when they may be facing the most difficult time in their lives, is a monumental responsibility. In an effort to provide CLSs with another tool to use in supporting children, we introduced a humanoid robot (NAO robot produced by Softbank Robotics) named MEDi (programmed with cognitive behavioral strategies) to a children’s hospital.

### Humanoid Robots

Humanoid robots are increasingly recognized for their potential to support health care in pediatric hospital settings [4,5]. One of the first studies published on the use of a robot during a medical procedure began in 2011 and was focused on MEDi. This study showed that MEDi, when programmed to distract children and teach them breathing as a coping strategy, reduced children’s pain and anxiety during vaccination [6]. This same robot also made children and their parents smile, helped children to cooperate during the procedure and to remember the robot more than the needle, and seemed to empower effective coping [7]. When MEDi was used to support children undergoing blood tests, parents reported that their children experienced reduced pain [8]. MEDi was then used with a variety of other medical procedures, such as intravenous starts, tube removal, and dressing change, with similar effects on pain and fear [9]. Other studies produced comparable results in various areas throughout the hospital using this robot [10,11]. Other studies have also suggested that children in a health care environment are enthusiastic about engaging with a humanoid robot [12], particularly children with autism [13,14].

This accumulating evidence of children’s enjoyment of humanoid robots and the ability of such robots to calm children’s pain, fear, and anxiety aligns well with the supportive role that CLSs have in providing medical procedural support to children to help reduce their distress. Indeed, the integration of a humanoid robot into CLSs’ daily practice is increasingly endorsed [15]. At the same time, a body of literature about the impact of humanoid robots on children’s hospital experiences is drawn from studies of the perspectives of parents and children. To our knowledge, no research has been conducted to capture the experiences of CLSs charged with incorporating humanoid robots into their day-to-day pediatric support practices. In this study, we conducted an in-depth examination of the experiences of CLSs using a humanoid robot in their daily work and our purpose is two-fold: (1) we aim to contribute to the understanding of the human-robot interface as part of supporting children in their hospital experiences, and (2) we will provide practice-relevant illustrations of the use of a humanoid robot in CLS practice. We present this study beginning with a description of the background of the CLS practice environment that provides the study context. We then provide a rationale for our narrative approach to conducting our study using semistructured interviews with CLSs, followed by a detailed illustration of CLSs’ experiences with using humanoid robots. We conclude with a discussion of our conceptual contribution and our practice-relevant recommendations.

## Methods

### Study Context

In 2014, 4 MEDi robots (NAO robots produced by Softbank Robotics) were introduced to Alberta Children’s Hospital, a 141-bed and 90-clinic tertiary care center seeing visits from 90,000 children per year, located in Western Canada. This introduction was based on research evidence of the impact of the robot on patients at this hospital. It became the first children’s hospital in North America, and perhaps in the world, to use robots for inpatient units and ambulatory clinics, tests and procedures, educational and orientation sessions (or *happy visits*), short or long admissions, and with any child where it seemed feasible. At any one time, any one or more of its 4 robots were used throughout the hospital for various purposes. The robot is 22.5 inches tall and weighs approximately 12 lb [16]. It is programmed with a variety of behaviors, such as telling stories based on themes of encouragement, playing interactive games to teach coping skills such as breathing, dancing to attract children’s attention to elicit smiles and laughter, and friendship behaviors such as fist bumps to build rapport. CLSs can also type text into the tablet that operates the robot to have it speak with animations in response to a child’s questions/comments. Pearson and Beran [17] provide a full description of MEDi behaviors. Since the introduction of MEDi in 2014, the team of CLSs regularly using MEDi shared many stories, experiences, and emotions with one another. It became evident that CLSs held compelling and detailed insights into how MEDi was affecting children, their parents, other health care professionals, and themselves. These accumulating and diverse anecdotal accounts added impetus to our plans to conduct this study.

### Study Design

To gain detailed insights into CLSs’ experiences with using MEDi to interact with children and their families, we used a narrative design. Narrative designs are built on the belief that people use stories—defined as consequential linkings of ideas or events [18]—to give others access to the richness of their experiences. We collected CLS stories about using MEDi by conducting semistructured individual interviews. The semistructured design of our interviews was used to keep interview conversations focused on experiences using MEDi while affording flexibility to follow the energy of participants by asking additional questions and prompting participants to more fully describe the experiences they considered interesting and important. Participants’ stories gave us access to narrative elements of their experiences using MEDi, such as settings, problems, actions, and resolution [19]. We were then able to bring individual participant stories side by side to draw out links and broader themes that tie stories together.

### Sample and Recruitment

We used purposive sampling to select the CLSs who were familiar with MEDi. At the time of recruitment (June to October, 2019), not all members of the child life team were regularly using MEDi in their work owing to responsibilities other than supporting medical procedures. In addition, as CLSs sometimes change roles, only those with the most extensive and recent experience with MEDi within the year before data collection were included in the study. This type of sampling enabled us to select participants according to their ability to inform our purpose of understanding their varied and detailed experiences [20].

The child life team was informed of the study by the team lead (fourth author, SB) during a staff meeting. An email was sent by the researchers to the 7 CLSs most experienced in working with MEDi, inviting them to participate in the study. The email invitation to participate contained information about the purpose and requirements of participation. All 7 CLSs agreed to be interviewed. All were women and had been CLSs for a period ranging between 1.3 and 29 years, with an average of 17 years of experience.

### Data Collection

Interviews were conducted in a private meeting room at the hospital where all the CLSs worked. Interviews were scheduled according to the CLS’s availability. Signed informed consent was obtained at the beginning of each interview. Participants were told that although their names would not be included in research reports, the small, in-depth nature of this study might make them identifiable, especially to readers who know the participants.

The first CLS participant interviewed was the second author (JP), given that she is also a CLS with 5 years of experience working with MEDi and was responsible for leading the integration of the robot into the child life team at this hospital. JP was interviewed by the first author (TB). This first interview and yielding data served as a pilot of the interview process during which the authors confirmed that the interview questions were meaningful and could be responded to within a standard 90-min research interview time frame. After this first interview, TB and JP together conducted the subsequent 6 interviews. These 6 interview participants worked with JP and were aware of TB’s role in introducing MEDi to their hospital workplace. Furthermore, TB and JP worked to maintain awareness of the separation between their own understanding and the descriptions given by participants by holding a stance of curiosity and openness. As such, we were attuned to various responses both between ourselves and the participants and across participants [21]. All interviews were audiotaped and transcribed verbatim by a research assistant (RA).

The interview duration ranged from 1 to 1.5 hours. The interview guide questions were open ended and invited story sharing and descriptions of actions, feelings, and thoughts about working with MEDi. Experiences were explored, and direct questions were sometimes asked for clarification of details [22]. Questions included asking about positive and negative experiences associated with using MEDi, how they use MEDi, others’ reactions to MEDi, the impact of MEDi on the hospital environment, and thoughts about the future use of MEDi.

### Ethical Considerations

JP’s collegial relationship with participants and TB’s introduction of MEDi to participants’ workplace required special consideration for all phases of the research. These relationships were disclosed to the university ethics board and to the manager at the hospital who approved this study. Furthermore, per our description of data collection, participants were reminded of the respective roles of TB and JP in relation to the topic being studied and told that their responses would not affect their daily responsibilities. We also assumed that as JP had been working with MEDi and her CLS colleagues for 5 years, negative experiences with MEDi would have been discussed between CLSs as these experiences occurred. However, we sought to create openness for participants to share negative experiences with using MEDi during interviews by explicitly requesting reflections about negative experiences. Moreover, to reduce concerns about encouraging positive responses and/or discouraging negative responses from participants during interviews, TB and JP made efforts to create a relaxed atmosphere, expressing their hope that the CLS participants would enjoy sharing their thoughts, challenges, and the range of feelings they have had in their work with MEDi. Finally, the length of the interview was adjusted according to participants’ energy and scheduling needs, and the participants were told that they could decline to answer any questions that made them feel uncomfortable.

### Data Analysis

A team of 4 analysts—the first 2 authors (TB and JP) and 2 RAs—analyzed all data. All 4 analysts have experience and education in interview data collection and analysis. The RAs had participated in another research project pertaining to the use of MEDi; thus, all 4 analysts were familiar with the subject of this study. We used a conventional thematic analysis approach to focus on generating themes from the content of stories shared by participants during interviews [23]. At the same time, this analysis is semideductive as themes were also shaped by the interview questions we posed to the participants. The third author (BL) has an extensive qualitative background and worked to enhance the credibility of the findings, consistent with the principle of triangulation of investigators. BL was not involved with the research until the analysis phase and used this distance to offset the closeness that the first 2 authors (TB and JP) had in the study.

Analysis began with authors independently listening to the recordings and reading and re-reading the transcripts. Following Corbin and Strauss [24], we independently identified concepts evident in each interview and made analytic notes (open coding). We then met to discuss these notes for each interview and then across interviews and were able to see each other’s perspectives and insights into and interpretations of each interview. We discussed detailed and frequently occurring responses that allowed us to draw out links and commonalities between codes, which we then organized into agreed-upon themes (axial coding). We deepened our analysis with the help of BL. BL is a seasoned qualitative researcher who was not involved in the implementation of this study and who comes from a different disciplinary background. As such, BL helped us triangulate investigator and disciplinary perspectives brought to bear on our analysis, which involved combining some themes into broader themes and resequencing some themes as components of the broader themes. We then conducted a member check by contacting all the participants to discuss the summary of our findings. TB and JP met with participants in a focus group format and invited them to reflect on their experiences, add more information, and share disconfirming thoughts. They confirmed that the findings represented their perspectives. Notes were maintained on these processes as an audit trail to further contribute to the trustworthiness of the findings.

## Results

### Themes

In this section, we most often refer to the robot as MEDi, as it is a trademarked name used by hospital staff and patients. The robot is also referred to as *he* rather than *it* to acknowledge that this former pronoun is more predominantly used by CLSs. In addition, in the interest of presenting our study concisely, we removed disfluencies in participant descriptions.

Through our thematic analysis, we generated 4 major themes. The first theme *incorporating a robot: navigating from fear to friendship* reflects the processes involved in learning to use a humanoid robot in a pediatric setting for therapeutic support. The second major theme is MEDi as a source of connection and support to children, and this theme comprises 3 subthemes: (1) MEDi as a welcoming and comforting presence, (2) MEDi as a friend, and (3) MEDi as encouraging and motivating. The third major theme is MEDi as an adaptable resource for CLSs, and this theme comprises 3 subthemes: (1) using MEDi to distract and teach, (2) using MEDi to facilitate challenging situations, and (3) using MEDi to facilitate collaboration within and beyond the CLS team. The fourth and final theme is working with the limits of MEDi.

### Incorporating a Robot: Navigating From Fear to Friendship

Of the 7 CLS participants interviewed, 4 had been on staff at the time of the robot’s introduction to the hospital. They described their initial feelings of uncertainty with CLS1 stating, “I didn’t really get what it was all about.” Feelings of initial fear and frustration as well as excitement and curiosity were shared. For example, CLS2 described how she had been *scared as shit*. Fear and uncertainty pertained to how to operate the robot—feeling nervous about breaking it and the frustration that occurred if the robot did not respond. These participants also reported a lack of understanding of how MEDi could be used with children. They felt embarrassed when MEDi did not work in front of a patient or his/her family. One participant had been determined not to use it, saying her initial response was, “…no not for me” (CLS4).

However, CLSs were emphatic in their descriptions of a turning point or a process of trial and error as they gained confidence in operating the robot and watched its impact. One of the CLSs said:

It took some time and a lot of conversations to figure out where and how it [MEDi®] would or where it would fit best.

CLS1

This CLS also explained that the children themselves showed ways in which the robot could be used:

…they sort of led the way in finding a way it [MEDi®] can be helpful and therapeutic to them.

CLS1

CLSs learned how to rely on their own skills of managing situations where the robot did not work properly. For example, CLS4 described becoming able to “…switch gears if I have to, whereas I think at first I was really intimidated by oh my gosh, if he breaks down…”

For her part, CLS4 described gaining familiarity saying:

So now I get it. I get how we can use him. It took me a while to get there, but I get that he can be used well in our profession.

CLS4

CLS4, the participant who had initially indicated that the robot was “not for me,” summarized her current thoughts about the robot stating, “I think we’re friends.”

Gaining comfort and confidence took investment from CLSs both to learn how to use the technology and open-mindedness in being willing to try something new. This frame of mind is ongoing and yields more possibilities. One of the CLSs noted:

…the more we give MEDi® to do, the more we can use him in different ways. It’s a process of growing and learning and figuring it out because there’s no manual on this. There’s no manual on what you do. You have the ability, skills, knowledge, and expertise but you have to figure out how to apply that teaching every time...it’s always a different day. So that’s the process of discovery with MEDi® too.

CLS6

### MEDi as a Source of Connection and Support for Patients

MEDi was described as a source of connection and support for children. We found that the depth of the support and connection afforded by MEDi varied, and we created subthemes to distinguish depths of support and connection according to (1) MEDi as a welcoming or comforting presence, (2) MEDi as a friend, and (3) MEDi as encouraging and motivating.

#### MEDi as a Welcoming and Comforting Presence

MEDi was portrayed by CLSs as a welcoming presence. CLS3 referred to MEDi as “…a friendly face” that contributes to “creating a positive experience in the hospital.” CLSs spoke of how MEDi could set the stage for future positive experiences:

I think there’s huge recognition now more than ever how important positive experiences at the hospital are for a child’s future visits here.

CLS7

I think he [MEDi®] opens doors to treatment in the future with or without him and kind of helps to...build those relationships and rapport.

CLS5

CLS1 shared a story of having ridden the elevator with MEDi in a sitting position on a cart. A child who had never seen MEDi before entering the elevator and immediately placed her hand on the cart and, on exiting the elevator, walked alongside for a while. CLS1 explained that MEDi seemed to be on this child’s level. CLS7 stated that she “…genuinely believed kids look at me as though he [MEDi^®^] is on the same level as them.”

During patient visits, the CLSs described MEDi as a comforting physical presence:

We know that a comfort position at the hospital is when a child sits between the parent’s lap, and MEDi® sits between the child’s lap and they hold on to him.

CLS7

CLSs noted that MEDi’s very presence seems to calm children:

...sometimes I’ve had it where he [MEDi®] doesn't say or do anything. He’s just there in the room and it seems to work. It seems to help which amazes me that he can just be sitting there and being hanging out there and he just seemed so friendly and comforting that he just has to be there you know.

CLS3

CLS7 noted that MEDi afforded a comforting source of continuity as she described a patient who was a *frequent flyer* who had had many visits to the hospital over an extended period. This frequent flyer patient would request to see MEDi during her visits. CLS1 also noted that MEDi could become part of children’s visits; however, CLS1 pointed out that the children did not necessarily actively interact with MEDi. Nevertheless, the presence of MEDi mattered:

...I’ve had situations where kids are completely happy with a MEDi® who’s turned right off. They’re just...oh this is so cool, and you know they just push MEDi® around on the cart and he's not even talking or anything.

CLS4

#### MEDi as a Friend

The nature of the support and connection that MEDi could provide to children was presented by CLSs as friendship. Some CLSs said that because MEDi greets children by name, a bond of friendship is formed:

I think she [4-year-old girl] formed this little bond with him [MEDi®].

CLS7

Another CLS recounted a patient reaction to the personalized connection MEDi offers:

…and he [MEDi®] knew her [the patient’s] name and he said, “congratulations” and just her eyes lit up …, “Oh my goodness! How does he know me? That’s so cool...My brother is gonna be so jealous.”

CLS5

The friendship style nature of connection is reinforced by children referring to MEDi as *he*, rather than *it*. Furthermore, friendship style connections persist even though children are well aware that MEDi is programmable and inanimate. One CLS described:

It’s funny how or interesting how kids will connect to him. In medical day treatment a kid was watching me program him. I showed her how we program him. We were picking things together. Yet, she still talked to him and was annoyed when he didn’t respond right away. And so, I think it’s interesting how they can connect even though they know it’s programmed from this tablet or that we’re using this tablet to help run him that they’re still engaged with him as like a person.

CLS5

Another CLS used the word love to characterize her sense of children’s fondness for MEDi:

The little four-year-old that I already talked about, I think that was it where I really saw the value of MEDi® and the collaboration that it offered with physio...she [the child] fell in love with MEDi®.

CLS2

#### MEDi as Encouraging and Motivating

MEDi was described as encouraging and motivating children to do activities they would not otherwise do. One of the CLSs presented MEDi as instilling courage:

And he does give kids courage. I think there’s a lot of kids we’ve seen that gain courage from seeing MEDi® do something and recognizing that they can do it too.

CLS7

A particularly tender moment was shared by one child, according to a CLS:

She [female patient] looked at her mom and she said, “MEDi® thinks I’m brave.”

CLS7

Children were described as seeming to implicitly trust MEDi and believe that words of encouragement that come from MEDi are sincere.

MEDi helped children manage during procedures that they had formerly found to be extremely difficult. CLS1 shared a story of a young boy with autism who would not sit on the dentist’s chair. After meeting MEDi, this boy agreed to sit on a chair with MEDi in his lap. The boy cooperated with all the dental procedures, and afterward, the mother blurted to CLS1, “That’s not my son,” as she could not believe that her son had allowed the dental assistant to touch and examine his mouth.

Another CLS expanded with a story of the surprise over the influence of MEDi expressed by health care staff; the staff had planned to sedate a boy for a procedure that entailed removing approximately 40 bumps that covered his body. The CLS described:

He [the boy] had played with MEDi® throughout the whole procedure...when I was leaving everyone who was involved was so impressed and the plan was to try it this time and then maybe go back to doing this procedure in the future under sedation. But then they called me a little while later and booked me for another date to come back with MEDi®.

CLS1

CLS2 shared a related story of a girl who would not respond to the physiotherapists who were encouraging her to sit up, build some core strength, and get out of bed. However, the child responded when the CLS went into this child’s room with MEDi:

Well that kid almost leaped out of her bed and she has tubes hanging out of every orifice. So, it was a challenge to slow her down, but then physio and I collaborated.

CLS2

Another CLS used the word *power* in explaining the surprising impact MEDi has:

…Because that [medical procedure] was such a hard one and it was right in the beginning when we really didn't know the power of MEDi® let’s say. The parent’s reaction that, “Oh my God, we’re actually going to get this [medical procedure] done. We’re going to get them through day surgery,” was phenomenal…that event actually opened up our eyes to what, how MEDi® can affect patients.

CLS6

One of the CLSs extended the idea of how MEDi affected patients as she described the impact of MEDi on her own children. She noted that her own children met MEDi when her son was in hospital:

He thinks it’s amazing that I get to work with a robot. My daughter wants to be a CLS when she grows up.

CLS7

### MEDi as an Adaptable Resource for CLSs

MEDi is an adaptable resource used by CLSs to achieve the therapeutic goals they set on the basis of their understanding of the child’s needs, the ability of MEDi, the parents’ behaviors, the needs of the health care staff, and their own expertise in child development. In other words, CLSs took many factors into account and used MEDi to help achieve their goals. Some CLSs always take MEDi with them to see children, whereas others stated that they first ask the parents if MEDi seems a good fit to their child’s interests. As CLSs worked to determine if and how children would find robot behaviors desirable, they explained how MEDi can be used in many situations and in different ways that allow them to be creative. We display CLSs’ use of MEDi according to the subthemes of (1) using MEDi to distract and to teach, (2) using MEDi to facilitate challenging situations, and (3) using MEDi to enhance collaboration.

#### Using MEDi to Distract and to Teach

CLSs described using MEDi to distract the child’s attention away from the medical procedure; helping the child to cope with anxiety, fear, and discomfort; and allowing the health care professional to complete the medical task more easily. One CLS spoke of the distinct distracting potential of MEDi:

...the child won’t look down won’t notice the poke, will be too busy watching MEDi®. He’s a great distraction ‘cuz unlike the iPad, he’s real and in front of you. He’s 3D. You can see him.

CLS3

When and how CLSs introduced MEDi in distraction for procedural support situations depended largely on the child’s age. When asked to use the robot with children aged 3 years and less, CLSs noted exercising additional caution knowing that in this stage of child development, children are often unable to separate fantasy from reality and may be afraid of the robot.

For older children and adolescents, CLSs described drawing on the robot’s technical attributes. One CLS spoke of how she used MEDi to engage a long-term teen patient:

He’s older so it’s less about the songs and dances at the age which he was really intrigued and captivated by kind of how it works - robots in general.

CLS5

Another CLS explained how she would tailor the intervention involving MEDi to the age of the patient:

At times, I’d introduce it to an older child and more from the scientific side approach...giving them the control over the tablet...using that teaching mode as their form of distraction...which is just as therapeutic...

CLS2

Although CLSs reported weighing countless variables and striving for the highest standards of professionalism, several noted that not every effort to incorporate MEDi as a distraction was successful. One CLS clearly described a difficult situation:

I think mom had met MEDi® and wanted us to use MEDi® but then MEDi® kind of did end up being that extra noise and confusion in the room...which sometimes happens where if he’s not distracting and he’s dancing...we needed more calm which is not all on MEDi® cuz I’m the one running MEDi®...In that situation it didn’t work, it just added more chaos and instead of that distraction piece or calm that we needed.

CLS5

According to CLS6, sometimes you realize, “MEDi^®^ is being one extra voice added on to the layer of voices in there …that it’s just not working out as a distraction or support.” In these cases, CLSs explained how they would shift to alternative forms of distraction or other coping strategies, debrief with families following the intervention, and adjust future plans for support accordingly.

As an alternative to distracting children from procedures, CLSs used MEDi to teach children specific coping strategies to use during procedures. In one particular application, MEDi instructs, demonstrates, and plays a game to encourage children to slowly inhale and exhale their breaths. Several CLSs mentioned this application. By learning these breathing strategies from MEDi, children can develop courage and confidence in dealing with procedures. CLS7 concisely described how this can work for children: “If MEDi^®^ can do it, I can do it too.”

CLSs also used MEDi to teach through play-based interventions. CLSs dressed MEDi as a patient and interacted with the robot in a child-friendly manner to explain why certain medical procedures were needed and what would be involved. CLS6 explained, “We do the vitals on MEDi^®^ first and then on the patient.” With MEDi cast in the role of patient, children would care for the robot in the way that the health care professional would care for them. MEDi-as-patient then responded with positive feedback about how gentle and caring the child had been. In this way, children can learn that the health care staff have positive motives even though sometimes they have to perform painful medical procedures.

#### Using MEDi to Facilitate Challenging Situations

CLSs described using MEDi in challenging medical situations such as those that can accompany supporting children with autism spectrum disorder (ASD). CLSs described how children with ASD could benefit in particular ways:

I definitely think that...kids on the autism spectrum love MEDi® because they can connect with him.

CLS3

Children with ASD may find it easier to negotiate their behaviors in interaction with a robot rather than in interaction with other people who tend to express a great deal of verbal and social information that may overwhelm the sensory processing systems of children with ASD. One CLS summed up this idea by saying:

I’ve noticed that children with autism tend to engage with the robot more than with me.

CLS1

CLS7 expanded on the idea of using MEDi in challenging situations as she described a patient who had an audiology examination. This patient’s grandmother brought her to her examination because there was stress in the family and that day was also the patient’s birthday. CLS7 described wanting to make sure the experience would be “super fun for her and we have the robot.” She brought MEDi to the examination area, and MEDi was carrying a balloon and a present for the patient. CLS summarized, “I think it just worked.” This CLS summarized how a good fit between what a child needs and what MEDi could provide can yield incredible results:

But I do think that first, when we pair him [MEDi®] up with the right kid, he can make them do just about anything.

CLS4

Although CLSs adapted their use of MEDi according to the needs of patients and used MEDi to distract, teach, and manage challenging situations, they also remained open to new possibilities. One CLS described:

I think of MEDi® as being another tool...when I’m trying to decide the best way to meet a family’s needs or the needs of a situation...I wonder if we could blow bubbles. I wonder if they’re allowed to go outside...I wonder if there’s a place here for MEDi®.

CLS4

Keeping MEDi’s potential in mind was conveyed by CLS3 who described MEDi as, “malleable and adaptable to whatever the situation needs him to be” and notes being often surprised. CLS3 initially thought MEDi was useful for certain children for specific reasons; however, then she began noticing that children would interact with MEDi in other ways that seemed to leave them comforted and relaxed. This CLS further explained, “So, I kept being surprised at different things throughout…,” noting that, “Kids can make him [MEDi^®^] what they need him to be and that’s what I was most amazed by him throughout getting to know him is that he’s not this cookie-cutter.” Indeed, the ways in which MEDi could be adapted made him a preferred resource distinct from others. One CLS said:

I would use MEDi® before other approaches now because I know I have MEDi®...and I’m not going to do [teach] deep breathing on my own.

CLS3

#### Using MEDi to Enhance Collaboration Within and Beyond the CLS Team

MEDi was discussed not only in terms of his therapeutic benefits for children but also for how he affected adults. MEDi was referred to as part of the CLS team. CLS7 said, “…it’s a collaborative effort and I think he's part of that team.” CLS7 drew a distinction between MEDi and other technology-based resources referring to how she and her colleagues, like the children, refer to MEDi as *he* rather than *it*:

I look at the iPad as an object that can provide distraction. Whereas I do tend to use MEDi® as more I guess, a part of our team...I can call him a he. I wouldn’t call my iPad a he.

CLS7

Indeed, in sharing stories of how MEDi affected patients, CLS3 even said that she *loves* MEDi.

MEDi being viewed as part of the team was evident when CLS4 spoke of the need to care and advocate for MEDi:

You have to have somebody who can look after him...he can’t just be left in a cupboard then people just grab him when they need him. He needs somebody to advocate for him and look out for him.

CLS4

MEDi also helped facilitate collaboration with colleagues beyond the CLS team, as illustrated by CLS2, who described working with colleagues from other departments of the hospital in efforts to optimize the benefits of using MEDi:

The cystic fibrosis team that I worked with, especially...she [healthcare staff] loved it when MEDi® came by…she got excited and where she got to the point of requesting MEDi® for certain kids when they do their morning assessments if we knew a certain child was going for blood work. She would tell the child about it saying, “You know, [the CLS is] around to help you with blood work today and she can go get MEDi®.” So, she would tell me and then we would make that happen in the morning. So, she was a great referral for that.

CLS2

Several CLSs recount how new working relationships were formed because of MEDi:

...in some of these clinic areas we weren’t very involved and our first involvement came to be because of MEDi®…the robot was the point of interest, caught people’s attention and then we started talking about ways in which we could integrate the robot into these areas.

CLS1

Many of the CLSs enthusiastically reported a unique collaboration with a particular physician and feelings of pride in this accomplishment:

I think the fact that [he] books his clinics around our schedule speaks to that.

CLS7

CLS4 noted potential of MEDi to provide a valuable distraction as she shared her impressions of working alongside this physician in this clinic with MEDi for the first time:

...I was just so blown away by what a great fit that was for all of these boys that were having some pretty uncomfortable surgeries while they were awake...For them to walk into an operating room and see a robot and [say] “what is that?” and then for us to be able to keep them distracted during all of that. ...to me, that’s super powerful…

CLS4

CLS7 spoke broadly about the collaborative influence of MEDi on the CLS reputation in the hospital as she indicated MEDi as having, “increased our popularity in general at the hospital” and that she feels like a *celebrity* because MEDi attracts so much attention and comedy. Indeed, CLS6 pointed out the energy that MEDi could catalyze among adults that extends to dancing with MEDi:

...we concentrate a lot on what MEDi® does for patients but it’s amazing how much MEDi® does for staff or parents...maybe we take it for granted a little bit, but you know having all of the staff watch you and they’re actually laughing or they’re doing the Gangnam Style [dance] with MEDi®. I think that speaks volumes of the effect that MEDi® could have on people I guess in general.

CLS6

### Working With the Limits of MEDi

CLSs described the limits of MEDi. Being unable to meet high expectations people held for MEDi and managing mechanical and operational difficulties were mentioned. CLSs described health care staff and patients as surprised at how MEDi seemed to surpass their expectations of what would typically be seen in a hospital. This could lead to high, difficult-to-meet expectations for what MEDi can do. MEDi could be viewed as a quick fix for any child who was anxious during a medical procedure. CLS1 explained that if a child was struggling during a procedure, he would be called *to come and fix it magically*.

Very nature of MEDi could contribute to conflicting expectations. As one of the CLSs noted:

It’s hard to put the words around it too because it’s not something that belongs in any other category–toy, person, tool.

CLS7

Indeed, MEDi could bring expectations of being able to perform any human function, such as responding to spontaneous conversation. When MEDi was unable to respond this way, people expressed disappointment. Children with repeated visits sometimes asked for more songs and games than MEDi was programmed to perform. For example, MEDi was able to play segments of some popular movie songs but was not continually updated as new movies were released. Thus, MEDi created high expectations and then was unable to meet additional expectations.

As with all machines, operating MEDi sometimes entailed technical difficulties. Because a tablet was used to operate MEDi through the network of a nearby router, the connection to the router was sometimes lost because of interfering signals from other machines. This problem was manageable once CLSs learned how to reconnect to the router. However, CLSs expressed that although MEDi is *kid-friendly*, it is not always *user-friendly*.

MEDi is designed and programmed to appear and act human-like, and most CLSs described MEDi in relational terms relative to patients and even relative to themselves. However, MEDi is a machine consisting of computers, wires, speakers, motors, and a variety of other parts. Machines break down or fail. CLSs expressed being frustrated, yet learning how to implement MEDi as a therapeutic tool, regardless; that is, they described how the breakdown can be presented to the child as a *human quality* in the same way that humans are not *always on our game*. They would also say, for example, that MEDi was not feeling well today and share this reflection with the child about not always feeling well, as a way of normalizing this experience.

## Discussion

### Principal Findings

Over several decades, traditional doctor-patient paternal approaches to health care have been moving toward a model of patient-engaged care [25]. Health care that takes into consideration the holistic needs of a patient, orients support toward those needs, and empowers children to feel brave—even in the most challenging medical situations—now represents the highest-quality health care experience possible [26]. Coming alongside this approach to health care is our introduction—almost 10 years ago—of a humanoid robot in a pediatric hospital. By examining stories about this implementation from the perspectives of the CLSs who regularly use the robot, we illustrate pediatric patient support that is provided at the level of the child and that we believe constitutes an exemplar of patient-engaged care.

Our evidence of patient-engaged care is in the form of 4 major themes of how CLSs experienced the integration of a robot into their daily practice of supporting children and families at a Western Canadian children’s hospital, and these themes represent a holistic, empowering approach to pediatric care. The first theme, navigating from fear to friendship, is reminiscent of an explorer in the process of discovering new territory. As the daily use of a humanoid robot in a hospital setting to provide support to children was innovative and not previously done at the time it was introduced at Alberta Children’s Hospital, there were no resources or previous learning to serve as a guide. Rather, CLSs took bold risks in trying to use the robot in various ways and types of medical situations, with patients having different backgrounds—never knowing what reactions would occur from patients, parents, and health care staff. It was a risky adventure in an emotionally charged environment.

As with any exploration into the unknown, the CLSs reported uncomfortable and even frightening feelings. These reactions are typical when learning something new, especially when presenting something new to an *audience* of children, parents, and health care staff who are facing a challenging medical event. We submit that patient-engaged care is enhanced when care providers join patients in taking risks. In this case, the CLSs who are encouraging children to be brave and face scary and painful medical challenges are also willing to take risk in trying a robot when the outcome is unknown.

Although we explored how CLSs’ perceptions of working with MEDi shifted over time, we did not ask specific questions about how they thought children’s reactions to repeat visits with MEDi may have changed. There were some suggestions (eg, children developing a bond over time and becoming more comfortable and the robot setting the stage for future positive experiences); however, these experiences need to be more thoroughly addressed in future research.

The second major theme about MEDi as a source of connection and support to children reflected how the robot could inspire comfort, friendship, encouragement, and motivation. These positive sentiments were suggested in a study on the same robot that interacted with children with diabetes [27]. Drawing on MEDi’s friendly appearance, CLSs brought MEDi into a relationship between themselves and the child. They used MEDi almost as an extension of themselves by having the robot speak encouraging phrases accompanied by expressive behaviors. A line of inquiry that emerges from this theme is whether CLSs select phrases and behaviors to play on the robot that they themselves would use. Alternatively, CLSs may create a unique identity in the robot and play the actions that the robot itself would seem to personify. What is clear is that CLSs worked in a patient-engaged manner to determine what children need to feel stronger and supported, and CLSs learned how to use the robot in ways that met those needs.

Perhaps the reason that the robot could be used as such an effective support is its ability to provide physical comfort. Even its mere presence can create a focal point for children to be distracted from negative feelings. Indeed, Alemi et al [28] provided some preliminary evidence that a robot may reduce children’s anger. MEDi’s size and human shape may invoke patient-engaged feelings of warmth and comfort. CLSs’ relational presentation of this human-like object can tantalize children’s imagination and spark their sense of connection with MEDi.

CLSs’ sense of delight in how children interacted with the robot seemed to motivate patient-focused professional energy on the parts of the CLSs. Although they used MEDi to provide support, such as they would with tools, for example, iPads, bubbles, and so on, they reported distinctly positive feelings associated with MEDi as they spoke of MEDi as a *he* and talked about MEDi as a member of the team. It is almost as if the adults saw the robot as more than a robot just as the children did.

CLSs described MEDi as an adaptable resource for use to distract, teach, facilitate challenging situations, and promote collaboration with health care staff. By situating the robot as expressing kindness, care, and affection toward children, children’s rapport and bonding with the robot created the platform from which a variety of strategies could be used. The result, as one CLS stated, was *to make them do just about anything*. They used MEDi in playful and instructive ways to empower children to cope with the distress of being in a hospital. They had the robot talk and act toward the child in any way they thought the child needed to the extent of personifying MEDi in different roles such as play partner, companion, or mentor. Although objects other than robots such as toys, dolls, and stuffed animals can also be used to provide support [29], the difference is that the robot (as controlled by the CLS) responds to what the child says and does. This special interactional experience affords creativity for CLSs. CLSs described extraordinary patient-engaged moments such as the robot celebrating a patient’s birthday with a gift and balloons on a day that anyone would feel overwhelmed by family and medical challenges. Not only did children experience these tender moments, the impact extended to health care professionals and parents as well. For example, how often is anyone seen dancing in a hospital?

Our final theme reflects the complexity of how CLSs are able to work with the limits of the robot. The surprise of meeting a robot created expectations and the desire for more human-robot interactions. Known as expectation discrepancy [30], people can experience disappointment when they realize a resource’s limited capabilities. CLSs may want to be aware of this potential outcome and preplan and creatively seek a variety of uses for MEDi so that MEDi’s impact can continue to be positive. For example, MEDi can be programmed with a variety of empirically supported psychosocial support strategies and relaxation techniques. As technology advances, more user-friendly programming capabilities can also enable CLSs to create any text and behaviors at the moment they wish to play them. In learning how to operate the robot, CLSs do not typically have training in using such a technologically sophisticated device. Thus, as more people become familiar with MEDi’s implementation in daily practice, this learning can be passed along to new users. As researchers with some familiarity of the experience using a humanoid robot for pediatric support, we too struggle to understand and put into words the contradiction of what type of entity the robot is. Perhaps what is more surprising though is that despite our ignorance, CLSs have, nevertheless, managed to resourcefully use this unusual tool as a means of supporting one of the most vulnerable populations in our society.

### Strengths and Limitations

As this study was conducted at only one hospital, the results may not be transferable to other hospital settings where the same or other robots are used. A strength in terms of transferability, however, is that all of the CLSs who had the most experience using the robot agreed to participate in this study. These results may, therefore, be relevant to other CLSs working with a robot. Furthermore, CLSs offer distinct insights that are rooted in the multidimensional experience inherent to the demanding role of the CLSs.

Our results are further limited given that interviews took place at one point in time and relied on recall, which is likely biased by memory. In addition, the familiarity between the researchers and the participants may have influenced responses in the interviews, despite our attempts to limit this effect. Thus, we see these results as suggesting patterns and themes to be explored further in future research. We recognize that despite deliberately deciding on approaching the data collection and analysis with open minds, our own social and cultural understanding of the CLS environment cannot always or easily be set aside. However, it is also true that the history and investment of the first 2 authors (TB and JP) in MEDi offers us a distinct position from which to understand and interpret participant meanings both *in the moment* during interviews and later as analysts.

### Conclusions

This is the first study to examine the professional life of CLSs working alongside a robot. At first glance, the themes may appear as surprising to the reader as the experiences were to the CLSs. Working with a humanoid robot for social interactions with a vulnerable population is not an everyday experience for most people. Our aim is to offer a glimpse into what new experiences may be created with the introduction of a humanoid robot in a children’s hospital. This study is a change in direction from our previous quantitative research examinations of the impact of the MEDi robot on children undergoing various medical procedures. Having obtained some empirical evidence of a reduction in patient distress [6-11], it seemed timely to next look at the nature of this experience through the eyes of the professionals who are orchestrating the operation of the robot. As CLSs continue to forge new ground, we will continue to come alongside with a research lens to present to the world what this strange new humanoid robot phenomenon is all about.

## Abbreviations

ASD: autism spectrum disorder
CLS: child life specialist
RA: research assistant
